# Bleeding From the Eye: An Unusual Presentation of Lacrimal Canaliculitis

**DOI:** 10.7759/cureus.20567

**Published:** 2021-12-21

**Authors:** Thomas Hickman Casey, Mary Sisley, William Saldana, Fraser S Peck

**Affiliations:** 1 Ophthalmology, Eastbourne District General Hospital, Eastbourne, GBR; 2 Ophthalmology, East Sussex Healthcare National Health Service (NHS) Trust, Eastbourne, GBR; 3 Ophthalmology, Bradford University, Eastbourne, GBR; 4 Pharmaceutical Medicine, Richmond Pharmacology, London, GBR

**Keywords:** lacrimal canaliculus, canaliculoplasty, chronic canaliculitis, lacrimal stones, actinomyces israelii

## Abstract

Chronic canaliculitis is an uncommon condition secondary to an infection of the lacrimal canaliculus, frequently caused by *Actinomyces israelii*. It is often misdiagnosed due to its symptoms mimicking more common pathologies and regularly fails to respond to antibiotics alone. Surgical intervention is the definitive treatment. We present a case of chronic canaliculitis with an unusual presentation.

## Introduction

Regurgitation of blood through the canicular system is a concerning symptom to patients. Chronic canaliculitis is a rare condition most frequently caused by infection. The most common causative organism is *Actinomyces israelii* [1]. These anaerobic Gram-positive, filamentous bacteria are normal commensal flora of the human digestive tracts. Diagnostic delay is often encountered due to the non-specific nature of symptomology. Presenting complaints are frequently attributed to more common pathologies such as chronic conjunctivitis, trauma or chalazion. Once these have been excluded, isolating the bleeding source should include exploration of the canicular system. Lacrimal stones can often be excised from within the lacrimal system in cases of chronic canaliculitis. Histopathological assessment of these stones shows characteristic features of sulphur granules with filamentous appearance seen under Grocott staining and suppurative granulomatous inflammation. These are specific signs of *A. israelii*. To the best of our knowledge, a case presenting with an expulsive haemorrhage has not been previously documented. This highlights the wide range of symptoms that chronic canaliculitis can present with, and that a diagnosis should always be considered, particularly in patients not responding to antibiotics.

## Case presentation

**Figure 1:**
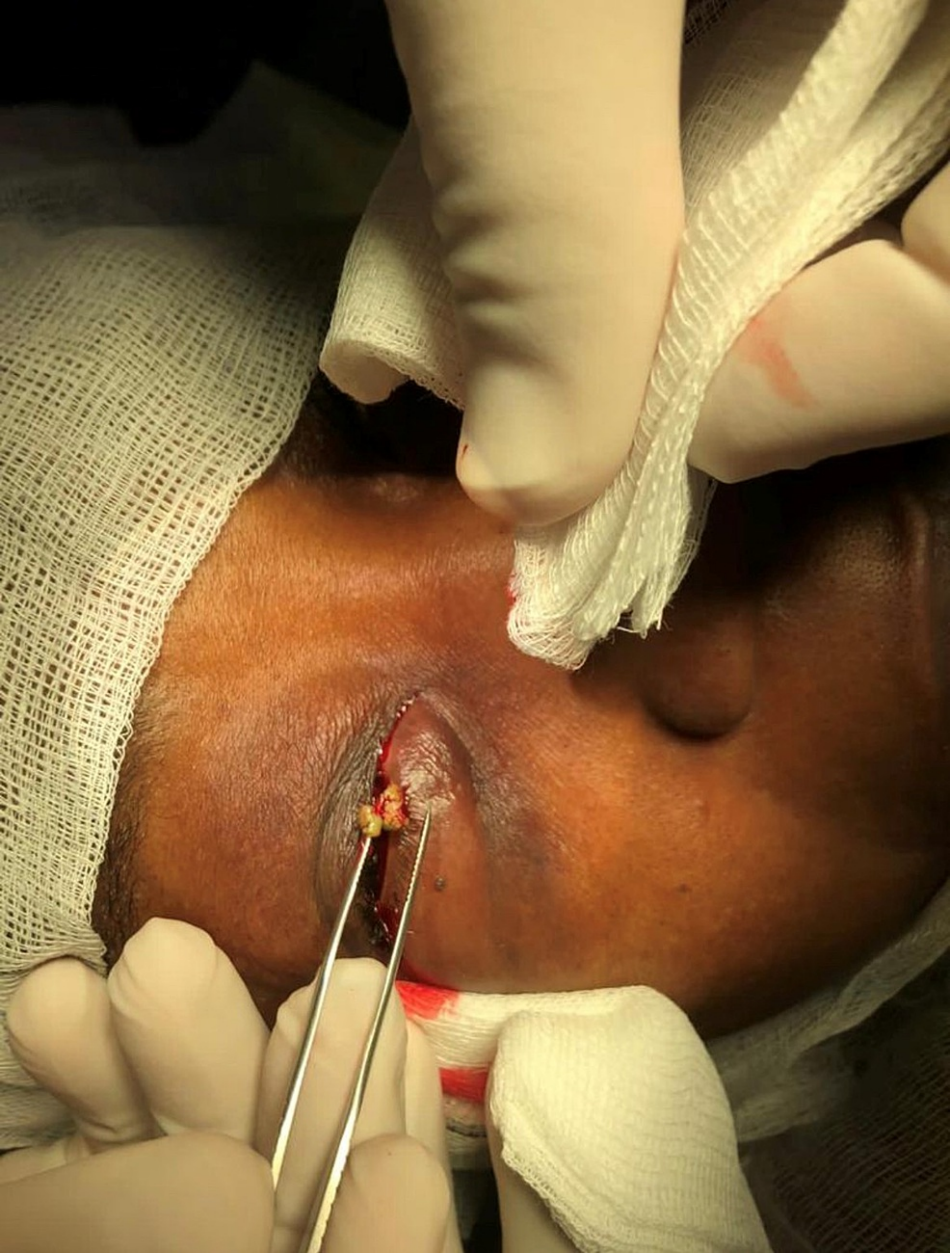
Photograph of lacrimal stones being removed during lacrimal system exploration

This is a photograph of five lacrimal stones retrieved at lacrimal system exploration of a 65-year-old patient (Figure 1). An otherwise fit and well female had been referred urgently to the eye clinic by her optometrist after she reported four episodes of “blood shooting out of her right caruncle”. She had recently undergone treatment with debridement and gland expression for meibomian gland dysfunction after a six-month history of bilateral upper and lower lid swelling.

She was diagnosed with right-sided canaliculitis with naso-lacrimal duct obstruction that had presented with painful “expulsive haemorrhage” from the right caruncle. Initial management included oral co-amoxiclav, oral doxycycline, fusidic acid, ofloxacin and chloramphenicol eye drops, but symptoms failed to resolve, with ongoing persistent discharge from the right lower punctum.

**Figure 2:**
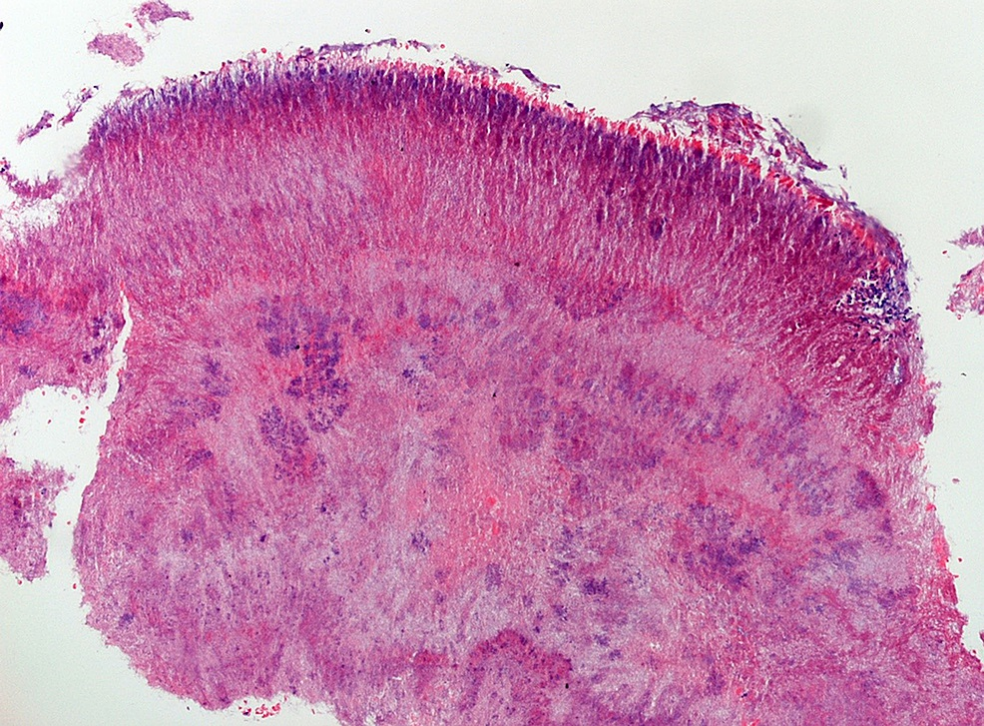
Tangled clumps of actinomycotic colonies with scanty surrounding fibrino-leukocytic exudate compatible with sulphur granules

**Figure 3:**
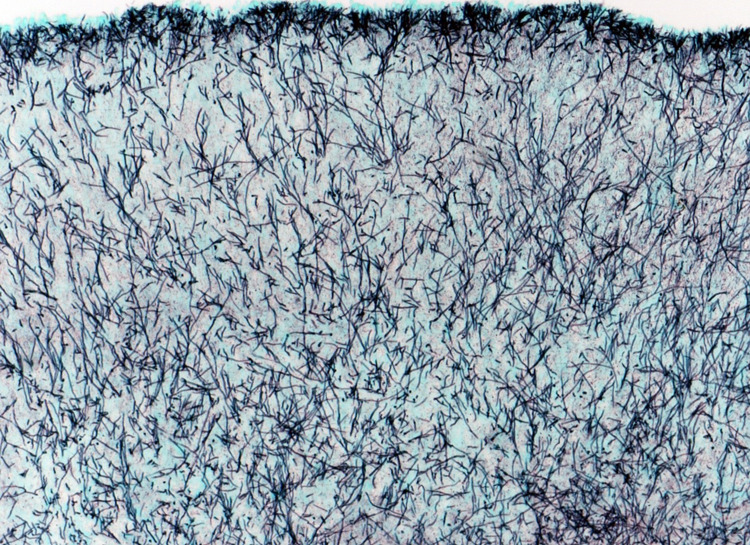
Grocott stain showing positive filamentous micro-organisms consistent with Actinomyces

A right-sided lacrimal canaliculotomy curettage was conducted due to a three-month failure of conservative management. Five lacrimal stones were retrieved at canaliculoplasty, biopsy and lacrimal system exploration (Figure 1), with the stones measuring between 1 and 5 mm in diameter. The surgery was uncomplicated and she experienced good post-operative recovery with oral amoxicillin and topical maxitrol. Histology of the retrieved lacrimal stones showed tangled clumps of Actinomycotic colonies, with scanty surrounding fibrino-leukocytic exudate compatible with sulphur granules (Figure 2). Grocott stain was positive for filamentous micro-organisms consistent with *Actinomyces* (Figure 3).

Two weeks post-operatively, no further discharge was noted. The tear film parameters were within the normal range on examination.

## Discussion

Canaliculitis traditionally presents with unilateral epiphora, chronic mucopurulent conjunctivitis, pericanalicular swelling and a “pouting” punctum [1]. “Yellow sulphur granules” at the punctum are pathognomonic for *Actinomyces canaliculitis* [1]. In the majority of cases, only a single duct is involved, with the lower lacrimal duct most frequently affected [2] and its prevalence is higher in women [3]. Chronic lacrimal canaliculitis accounts for 2% of lacrimal duct disease [4]. Due to its classical symptoms mimicking more common pathologies such as chronic conjunctivitis, chalazion, hordeolum or chronic dacrocystitis [5], diagnosis is often delayed and the mean duration of these symptoms until the time of diagnosis is 10 months [6].

Initial treatment options typically involve a course of topical antibiotics such as fluoroquinolone; however, this is often ineffective due to chronically colonized concretions [4]. Surgery with a canaliculotomy and curettage of these concretions or punctoplasty is frequently needed as a curative measure.

*Actinomyces* spp. is a Gram-positive anaerobic bacterium that is a normal commensal in the gastrointestinal and female genital tracts [7]. It is primarily an opportunistic, endogenous infection that can spread to the lacrimal system either directly or indirectly via contaminated oral secretions. There are at least 30 different species of *Actinomycoses*, with *A. israelii* the most common pathogen found in patients [8]. Its ability to form biofilms also adds to the difficulty in resolution with antibiotics alone [9].

In the presented case, there is no obvious predisposing cause for the *Actinomyces* infection to spread. A review of the literature suggests previous trauma, old age, living in a humid climate and female sex [10] as risk factors.

## Conclusions

Lacrimal canaliculitis is an uncommon condition with its symptoms often mimicking more frequently seen pathologies. It is most frequently caused by *A. israelii* and requires a long course of antibiotics, and often surgery is the definitive treatment. This case showcases an unusual presentation of this condition but the diagnosis should always be considered in patients who are refractive to antibiotics alone.
